# The acute diuretic effect of an ethanolic fraction of *Phyllanthus amarus* (Euphorbiaceae) in rats involves prostaglandins

**DOI:** 10.1186/s12906-018-2158-0

**Published:** 2018-03-15

**Authors:** Alain N’guessan Yao, Mamadou Kamagaté, Augustin Kouao Amonkan, Philippe Chabert, Fidèle Kpahé, Camille Koffi, Mathieu N’goran Kouamé, Cyril Auger, Séraphin Kati-Coulibaly, Valérie Schini-Kerth, Henri Die-Kakou

**Affiliations:** 1 Department of Pharmacology, UFR-SMA, Félix Houphouët-Boigny University, 01 BP V 166 Abidjan 01, Abidjan, Côte d’Ivoire; 2 Laboratory of Nutrition and Pharmacology, UFR-Biosciences, Félix Houphouët-Boigny University, Abidjan, Côte d’Ivoire; 3 UMR CNRS 7213, Laboratory of Biophotonics and Pharmacology, Faculty of Pharmacy, University of Strasbourg, Illkirch, France

**Keywords:** *Phyllanthus amarus*, Diuresis, Electrolytes, Prostaglandins

## Abstract

**Background:**

*Phyllanthus amarus* (Schum & Thonn)*,* a plant belonging to the family of Euphorbiaceae is used in Ivorian traditional medicine to treat cardiovascular disorders such as hypertension. However, although this plant has been described as a diuretic agent, the underlying mechanism remains unclear. Therefore, the aim of the present study was to investigate the mechanism action of diuretic effects of an ethanolic fraction of *Phyllanthus amarus* (EFPA) in rats.

**Methods:**

Effects of EFPA on urinary excretion were carried out for doses ranging from 5 to 80 mg/kg given by intraperitoneal injection (i.p.) and compared with that induced by furosemide (5 mg/kg) after 8 h. Thereafter, the diuretic activity of EFPA was also evaluated in the presence of indomethacin (5 mg/kg, i.p*.*) in order to determine the involvement of prostaglandins, after 24 h.

**Results:**

Between 5 and 80 mg/kg, EFPA induced a significant urinary excretion. The profile of urinary excretion showed that after 2 h, the highest dose of 80 mg/kg induced a urinary volumetric excretion (UVE), which was similar to that induced by furosemide. After 24 h, EFPA at 10 mg/kg increased significantly UVE, Na^+^ (43 mEq) and Cl^¯^ (97 mEq) urinary excretions without promoting kaliuresis. In rats pretreated with indomethacin, the urinary excretion and the natriuretic response of EFPA were significantly reduced.

**Conclusion:**

Altogether, this study has shown that EFPA promotes a significant urinary excretion of water and Na^+^, confirming its diuretic activity. Moreover, the increased diuresis could be attributed, at least in part, to the involvement of prostaglandins.

**Electronic supplementary material:**

The online version of this article (10.1186/s12906-018-2158-0) contains supplementary material, which is available to authorized users.

## Background

The World Health Organization (WHO) encourages developing countries from the tropical area to develop therapeutic alternatives in the management of priority and emerging diseases such as high blood pressure and diabetes [1]. This strategy involves exploitation of the potential medicinal plants from the local pharmacopoeia. In this context, many studies on traditional medicine are undertaken in African countries against hypertension [2].

In Côte d’Ivoire, several traditional herbal preparations are used by the local population to treat hypertension, among them, *Phyllanthus amarus* (Schum & Thonn)*,* a plant belonging to the family of Euphorbiaceae [3]. This plant has various applications in traditional medicine throughout the world [4, 5]. In the Ayurvedic medicine, decoctions of the whole plant are used to treat malaria [6], and skin disorders [7]. In traditional West African pharmacopoeia, particularly in Nigeria, the administration (per os) of decoctions of leaves and seeds extracts are used to treat diabetes [8].

Pharmacological studies in mice and rats have shown that *Phyllanthus amarus* has antiviral [5], hepatoprotective [9, 10] and hypoglycemic activities [11]. A previous study [12] has suggested that *Phyllanthus amarus,* described as diuretic agent*,* might be of interest in the treatment of hypertension. The antihypertensive activity of *Phyllanthus amarus* has been related to stimulation of muscarinic receptors leading to the formation of endothelium-derived nitric oxide (NO) and also to blocking of calcium channels [13, 14]. Moreover, Phyllanthus species have been shown to have several beneficial effects on the vascular function. Phyllanthin and hypophyllanthin, two major compounds from *Phyllanthus amarus,* decreased vascular tone by endothelium-independent mechanisms via the blockade of Ca^2+^ entry into the vascular smooth muscle and inhibition of phenylephrine-induced Ca^2+^ release from the endoplasmis reticulum [15]. *Phyllanthus acidus* also inhibited vascular tone in aortic rings by acting directly on the smooth muscle and/or by releasing NO from the endothelium [16]. Moreover, 6 weeks after oral administration of an n-butanol extract of the leaves of *Phyllanthus acidus,* a decreased contractile response to phenylephrine and an enhanced endothelium-dependent relaxation to acetylcholine were observed in aortic rings of middle-aged male rats [17].

Furthermore, several studies have shown that natural products such as plant extracts from *Hibiscus sabdariffa* and *Vepris heterophylla* have diuretic properties, which might contribute to regulate blood pressure [18, 19]. It is well known that diuretics such as thiazides play a key role in the management of hypertensive diseases. They are most often associated with classic antihypertensive drugs such as angiotensin II type 1 receptor (AT1R) blockers to optimize blood pressure control. In this case, the role of diuretics is to potentiate the therapeutic effect of AT1R blockers by inducing a significant renal elimination of sodium and water.

The evaluation of the pharmacological effects of a *Phyllanthus amarus* aqueous extract administered intravenously in normotensive rabbits has shown that at doses ranging from 4.5 to 71.7 mg/kg, the extract induced a transient decrease in mean arterial blood pressure of about 4.8 ± 0.8 to 14.0 ± 0.6 mmHg [14]. The ethanolic fraction was the most potent fraction inducing about a 11.5 ± 0.5 mmHg reduction of mean arterial blood pressure at 15 mg/kg [14]. In addition, it seemed to be the most potent extract for diuresis, however the mechanism action of these diuretic effect remain unclear [20]. The present study has evaluated the possibility that an ethanolic fraction of *Phyllanthus amarus* has a diuretic effect, which might contribute to reduce blood pressure.

## Methods

### Plant material

The whole plant of *Phyllanthus amarus* (Euphorbiaceae) was collected in the district of Cocody (Abidjan, Côte d’Ivoire) in April 2014. This plant has been authenticated by an expert in Botany (Professor Ake-Assi Laurent) at the *Centre National de Floristique* (UFR-Biosciences, Félix Houphouët-Boigny University, Abidjan, Côte d’Ivoire) where the voucher specimen was recorded under No. 3, 141 and 248.

### Extraction procedure

The ethanolic fraction of *Phyllanthus amarus* (EFPA) was prepared as previously described with minor modifications [14]. Briefly, the whole plant was harvested, washed, and extracted in boiling distilled water for 30 min at ratio of 500 g of plant for 1 l. The decoction was filtered and then lyophilized to obtain a powder of aqueous extract of *Phyllanthus amarus* (11.72 g from 1 kg of plant). 3 g of lyophilisate were then dissolved in 250 ml of a 70% ethanol solution and suspended into a separating funnel for 12 h. The upper phase was separated and dried using a rotary evaporator (Büchi) to obtain powder of ethanolic fraction of *Phyllanthus amarus* (EFPA). Extraction procedure was repeated 5 times and, the final yield of EFPA preparation was 0.26%.

### Pharmacological studies

#### Animal

Male Wistar rats weighing between 180 and 250 g were used. Animals were randomly assigned 6 rats per group, including control, Furosemide (5 mg/kg), EFPA at different doses (5, 10, 20, 40 and 80 mg/kg), and EFPA (10 mg/kg) + indomethacin (5 mg/kg). They were bred in the animal house of the *Ecole Normale Supérieure* (Abidjan). Before starting the experiments, rats were isolated and acclimated for 14 days. They were maintained under standard laboratory conditions (25 ± 2 °C) with dark and light cycle (12/12 h) and had free access to a standard dry pellet diet and water ad libitum. Experiments were performed in accordance to the Guidelines for experiments involving animals [21, 22].

#### Evaluation of the diuretic activity

Diuretic activity was determined by the method described by Kau et al. (1984) with some modifications [23].

In the first step of experiments, the evaluation of the effects of different doses of the EFPA extracts on urinary excretion was carried out for doses ranging from 5 to 80 mg/kg body weight i.p. after an 8 h treatment period. The effect of *Phyllanthus amarus* extract was compared with that induced by Furosemide (5 mg/kg, i.p.), used as a reference loop diuretic drug. The doses evaluated were below the maximal tolerated doses of *Phyllanthus amarus* administrated i.p. [24].

Thereafter, the diuretic effect of EFPA (10 mg/kg) was evaluated after 24 h in the presence of indomethacin (5 mg/kg), a cyclooxygenase inhibitor, in order to evaluate the role of prostaglandins. Indomethacin was administered i.p. to rats 1 h prior to the administration of EFPA.

The administration of each test substance was preceded by fluid overloading with 50 ml/kg of water per os. Rats were placed individually in metabolic cages. Urine volumes were collected over either 8 or 24 h periods and were used to determine the urinary volumetric excretion (U.

VE) from the ratio of the volume of urine excreted and the volume of fluid overloaded. The urine volumes expressed as mL/100 g were calculated based on body weight of rats and urinary volumetric excretion values are expressed as a percentage of the initial hydric overload (50 mL/kg). At the end of the 24 h period, urine samples were collected and aliquoted into eppendorf tubes. Animals were anaesthetized by intraperitoneal injections of pentobarbital (50 mg/kg). The thoracic cavity was opened by surgical operation and blood was taken directly by heart puncture, put into heparinized tubes and, thereafter the plasma was obtained by centrifugation. Finally, animals were sacrificed by exsanguination. Both urine and plasma aliquots were stored at − 80 °C for subsequent biochemical analysis.

#### Analytical procedures

In order to determine the electrolyte content in plasma and urine samples, several techniques were used. The determination of sodium (Na^+^) and potassium (K^+^) contents was done by photometry, chlorine (Cl^−^) and urea concentrations by a colorimetric technique, and the calcium concentration by quantitative CPC method (o-cresolphthalein complexone). The creatinine content was estimated by the kinetics method.

#### Drugs

Furosemide was from Tocris Bioscience (Abingdon, UK) and indomethacin from Sigma (Saint Quentin Fallavier, France).

### Statistical analysis

Data are expressed as mean ± SEM. Statistical analysis of data was performed using GraphPad Instat software (Microsoft, San Diego, California, USA) and Graph Pad Prism software (Microsoft, San Diego, California, USA). Statistical analyses were assessed using one-way or two-way analysis of variance (ANOVA) followed by Bonferroni’s post- test when applicable. Statistical significance was considered at *p* < 0.05.

## Results

### Effect of EFPA on urinary excretion

**Fig. 1:**
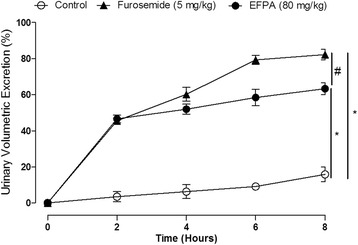
Evolution of the urinary volumetric excretion induced by highest dose of EFPA (80 mg/kg*)* and Furosemide (5 mg/kg), in rats. The urinary volumetric excretion values are expressed as a percentage of the initial hydric overload (50 mL/kg). Data are given as means ± SEM of 6 different experiments. Statistical analyses were assessed using two-way analysis of variance (ANOVA) followed by Bonferroni’s post- test. *p < 0.001 versus control, and ^#^*p* < 0.05 versus EFPA

Intraperitoneal administration of EFPA at doses ranging from 5 to 80 mg/kg body weight induced significant urinary excretion in a dose-dependent manner compared to the control group (*p* < 0.001; Table 1; Additional file 1: Figure S1). After 8 h, urinary excretion ranged from 41.2 ± 3.3% to 63.3 ± 3.2%, respectively (Table 1; Additional file 1: Figure S1). Urinary volumes excreted by the highest dose of EFPA (80 mg/kg) and 5 mg/kg of Furosemide were time dependent and significant compared to that of the control group (*p* < 0.001; Fig. 1). After 2 h, Furosemide and EFPA caused similar urinary excretion, whereas at 8 h, the effect of Furosemide was significantly greater than that of EFPA (82.2% ± 3.0 versus 63.3 ± 3.2%, respectively at 8 h; Fig. 1).

**Table 1:** Urinary excretion induced by increasing doses of EFPA in rats

Groups	Urine volume (mL/100 g)				Urinary volumetric excretion (%)			
	2 h	4 h	6 h	8 h	2 h	4 h	6 h	8 h
Control	0.14 ± 0.09	0.20 ± 0.07	0.50 ± 0.04	0.70 ± 0.05	2.70 ± 2.83	3.80 ± 1.10	8.76 ± 0.80	13.62 ± 0.92
EFPA (5 mg/kg)	0.84 ± 0.11^*^	1.49 ± 0.14^*^	1.73 ± 0.20^*^	2.09 ± 0.17^*^	16.61 ± 2.01^*^	29.40 ± 3.02^*^	34.20 ± 3.64^*^	41.20 ± 3.32^*^
EFPA (10 mg/kg)	1.14 ± 0.13^*^	1.82 ± 0.11^*^	2.21 ± 0.15^*^	2.80 ± 0.20^*^	22.92 ± 2.60^*^	36.50 ± 2.53^*^	44.20 ± 3.80^*^	55.94 ± 3.40^*^
EFPA (20 mg/kg)	1.67 ± 0.15*	2.06 ± 0.16^*^	2.34 ± 0.10^*^	2.86 ± 0.12^*^	33.30 ± 4.10^*^	41.23 ± 2.90^*^	46.80 ± 2.70^*^	57.10 ± 1.90^*^
EFPA (40 mg/kg)	2.02 ± 0.11*	2.34 ± 0.10^*^	2.51 ± 0.18^*^	3.09 ± 0.13^*^	40.60 ± 1.70^*^	47.12 ± 2.83^*^	50.53 ± 3.80^*^	60.20 ± 2.90^*^
EFPA (80 mg/kg)	2.33 ± 0.12^*^	2.60 ± 0.13^*^	2.92 ± 0.13^*^	3.16 ± 0.15^*^	46.61 ± 2.10^*^	51.90 ± 2.73^*^	58.41 ± 1.91^*^	63.34 ± 3.24^*^

The urine volumes expressed as mL/100 g were calculated based on body weight of rats and urinary volumetric excretion values are expressed as a percentage of the initial hydric overload (50 mL/kg). Data are given as means ± SEM of 6 different experiments. Statistical analyses were assessed using two-way analysis of variance (ANOVA) followed by Bonferroni’s post- test. *p ˂ 0.001 versus control

### Effect of indomethacin on EFPA-induced diuresis and excretion of electrolytes

**Fig. 2:**
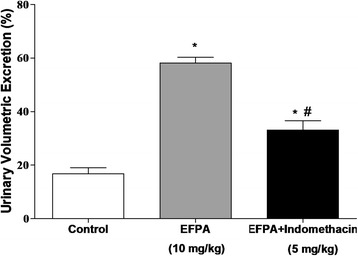
Inhibitory effect of indomethacin on urinary volumetric excretion induced by EFPA in rats. Rats were treated over 8 h period with a single dose of either vehicle (control group), EFPA (10 mg/kg) or EFPA (10 mg/kg) + indomethacin (5 mg/kg, 1 h pretreatment) administered i.p. Data are given as means ± SEM of 6 different experiments. Statistical analyses were assessed using one-way analysis of variance (ANOVA) followed by Bonferroni’s post- test *p < 0.05 versus control, and ^#^*p* < 0.05 versus EFPA

After 24 h, EFPA at the dose of 10 mg/kg increased significantly the urinary volumetric excretion (58.1 ± 2.15%; *p* < 0.05; Fig. 2), compared with that of the control group (16.8 ± 2.29%; *p* < 0.05; Fig. 2). In presence of indomethacin, the urinary volumetric excretion of EFPA was significantly reduced, amounted to 33.1 ± 3.5% (*p* < 0.05; Fig. 2), however, it remained greater than that of the control group (*p* < 0.05; Fig. 2).

After 24 h, EFPA (10 mg/kg) induced a significant urinary excretion of sodium (90.0 ± 4.7 mEq/L) compared to that of the control group (70.50 ± 6.1 mEq/L; Table 2; Additional file 2: Figure S2). In rats pretreated with indomethacin (5 mg/kg), EFPA-induced natriuresis was significantly reduced to about 43.5 ± 4.4 mEq/L (*p* < 0.05; Table 2; Additional file 2: Figure S2). In addition to sodium excretion, EFPA induced also a significant urinary excretion of Cl^−^ from 81.2 ± 3.1 mEq/L to 97.3 ± 2.9 mEq/L, which however was not affected by indomethacin (96.7 ± 4.1 mEq/L; Table 2; Additional file 2: Figure S2). EAPA also caused a significant reduction in the urinary excretion of K^+^ compared with that of the control group (52.2 ± 2.0 mEq/L versus 36.7 ± 1.6 mEq/L, respectively in the control and EFPA group), but in the presence of indomethacin, excretion of K^+^ was increased (61.0 ± 2.4 mEq/L; Table 2; Additional file 2: Figure S2). The EFPA treatment did not significantly affect calciury in rats (1.8 ± 0.1 versus 1.7 ± 0.1 mEq/L in the control and EFPA group, respectively), also in presence of indomethacin (3.1 ± 0.2 mEq/L; Table 2; Additional file 2: Figure S2).

**Table 2:** Inhibitory effect of indomethacin on urinary electrolyte excretion induced by EFPA in rats

Groups	Urinary concentrations (mEq/L)			
	Na^+^	Cl^−^	K^+^	Ca^2+^
Control	70.50 ± 6.05	81.20 ± 3.10	52.20 ± 2.01	1.80 ± 0.10
EFPA (10 mg/kg)	90.00 ± 4.70^*^	97.33 ± 2.90^*^	36.70 ± 1.63^*^	1.73 ± 0.10
EFPA (10 mg/kg) + Indomethacin (5 mg/kg)	43.50 ± 4.43^*,#^	96.70 ± 4.10^*^	61.00 ± 2.42^*,#^	3.10 ± 0.20

Rats were treated over a 24 h period with a single dose of vehicle (control group), EFPA (10 mg/kg) or EFPA (10 mg/kg) + indomethacin (5 mg/kg, 1 h pretreatment) administered i.p. Data are given as means ± SEM of 6 different experiments. Statistical analyses were assessed using two-way analysis of variance (ANOVA) followed by Bonferroni’s post- test. *p < 0.05 versus control, and ^#^*p* < 0.05 versus EFPA

### Effect of EFPA on plasma electrolyte levels

Control group and rats treated with EFPA (10 mg/kg for 24 h) had similar natremia (114.7 ± 5.5 and 125.5 ± 4.0 mEq/L, respectively; Table 3; Additional file 3: Figure S3), this effect was not affected in presence of indomethacin (119.3 ± 4.4 mEq/L; Table 3; Additional file 3: Figure S3). The plasma chlorine level observed in rats following the EFPA treatment was 102.0 ± 4.3 mEq/L, similar to that observed in the control group (104.3 ± 3.9 mEq/L), and not affected by indomethacin (103.3 ± 3.4 mEq/L; Table 3; Additional file 3: Figure S3). The potassium level was significantly reduced after EFPA treatment (70.5 ± 2.9 versus 54.7 ± 2.8 mEq/L; respectively in the control and EFPA group), this effect was not affected by indomethacin (Table 3; Additional file 3: Figure S3). The plasma calcium level following EFPA treatment was similar to that of the control group (5.2 ± 2.6 mEq/L and 5.4 ± 2.5 mEq/L, respectively) and this response was not affected by indomethacin (5.1 ± 2.5 mEq/L; Table 3; Additional file 3: Figure S3).

**Table 3:** Inhibitory effect of indomethacin on plasma electrolyte level induced by EFPA in rats

Groups	Plasma levels (mEq/L)			
	Na^+^	Cl^−^	K^+^	Ca^2+^
Control	114.70 ± 5.51	104.33 ± 3.90	70.50 ± 2.90	5.40 ± 2.52
EFPA (10 mg/kg)	125.50 ± 4.00	102.00 ± 4.32	54.70 ± 2.80^*^	5.21 ± 2.60
EFPA (10 mg/kg) + Indomethacin (5 mg/kg)	119.33 ± 4.43	103.33 ± 3.41	50.83 ± 5.10^*^	5.13 ± 2.54

Rats were treated over a 24 h period with a single dose of either vehicle (control group), EFPA (10 mg/kg) or EFPA (10 mg/kg) + indomethacin (5 mg/kg, 1 h pretreatment) administered i.p. Data are given as means ± SEM of 6 different experiments. Statistical analyses were assessed using two-way analysis of variance (ANOVA) followed by Bonferroni’s post- test. *p < 0.05 versus control, and ^#^*p* < 0.05 versus EFPA

### Effect of EFPA on renal function

After 24 h, the EFPA treatment (10 mg/kg for 24 h) affected slightly but not significantly the plasma concentration of urea and creatinine in rats, both responses where significantly increased in presence of indomethacin (Table 4).

**Table 4:** Effects of EFPA (10 mg/kg) and EFPA (10 mg/kg) + indomethacin (5 mg/kg, 1 h pretreatment) administered i.p. on plasma levels of creatinine and urea

Groups	Control	EFPA (10 mg/kg)	EFPA (10 mg/kg) + Indomethacin (5 mg/kg)
Creatinine (μmol/L)	94.28 ± 7.80	88.40 ± 4.56	110.47 ± 6.75^#^
Urea (mmol/L)	4.08 ± 0.38	3.29 ± 0.43	5.83 ± 0.58^*, #^

Data are given as means ± SEM of 6 different experiments. Statistical analyses were assessed using one-way analysis of variance (ANOVA) followed by Bonferroni’s post- test *p < 0.05 versus control, and ^#^*p* < 0.05 versus EFPA

## Discussion

The present findings indicate that an ethanolic fraction of *Phyllanthus amarus* (EFPA) at doses ranging from 5 to 80 mg/kg, significantly induced diuresis during an 8 h period. The profile of urinary excretion showed that after 2 h, at a dose of 80 mg/kg, EFPA induced approximately the same UVE than that of 5 mg/kg of Furosemide. However, after 8 h, the urinary excretion of Furosemide was greater (82%) than that induced by the EFPA (63%). Thus, these observations indicate that the diuretic effect of EFPA is relatively strong and sustained [25], as indicated by about 58% diuretic effect of EFPA at 10 mg/kg after 24 h.

The effect of diuretic substances is commonly associated with the urinary excretion of electrolytes. The present findings indicate that EFPA induced important urinary excretion of sodium (43 mEq) in consistent with previous observations showing that a preparation of the whole plant of *Phyllanthus amarus* caused significant urinary sodium excretion in hypertensive humans [12]. Furthermore, the EFPA-induced natriuresis was associated with urinary excretion of chlorine (97 mEq) without promoting excretion of potassium. In addition, a leaf extract of *Ficus exasperata* (50 mg/kg, per os) also significantly decreased the plasma sodium level without promoting kaliuresis in rats [26].

Preliminary phytochemical analysis of EFPA revealed the presence of alkaloids, polyphenols, terpenes and sterols [14]. The diuretic effect of EFPA could be most likely related to these secondary active metabolites. Indeed, polyphenolic compounds, triterpenoids and saponins have been described to contribute to the diuretic effect of several plant extracts including *Musanga cecropioides, Viscum angulatum*, *Hibiscus sabdariffa* and *Nigella sativa* [27–30].

Furosemide, a highly effective loop diuretic induced considerable loss of water associated with an urinary excretion of sodium, chlorine and potassium. However, previous reports have also indicated that Furosemide did not affect significantly the efflux of potassium in normal rats [31, 32]. Thus, the EFPA treatment seems to induce a similar diuretic effect as that of Furosemide, without a loss of potassium**.** The main mechanism associated with loop diuretics is linked to inhibition of the co-transport system Na^+^/K^+^/2Cl^−^ of the ascending limb of the loop of Henle [33]. Thus, EFPA could possibly act upon this part of the nephron to exert an inhibition of sodium chloride reabsorption and, hence, inducing a significant urinary elimination of water. Moreover, the diuretic effect of EFPA could also be related to other mechanisms such as affecting prostaglandin E_2_ (PGE_2_) formation, which regulates kidney function in the distal nephron by stimulating excretion of sodium via inhibition of vasopressin-stimulated water and sodium absorption in the collecting duct [34, 35]. In addition, PGE_2_ has been shown to play a key role in reducing vascular tone, and, hence, increasing renal blood flow leading to an increase of water exchange and transmembrane electrolyte, and an increased glomerular filtration rate [36, 37]. PGE_2_ is synthesized by the cycloxygenase COX-1 and COX-2 located in the cortical zone of the renal tissue. Previous studies have indicated that inhibition of COX-1 and COX-2 with indomethacin resulted in retention of sodium and water associated with hypertension and edema in rats [34]. The present study indicates that treatment of rats with indomethacin significantly reduced the natriuretic and the diuretic responses of EFPA. These findings suggest that the diuretic properties of *Phyllanthus amarus* can be partially attributed, at least in part, to the involvement of diuretic prostaglandins. A similar mechanism has also been involved in the diuretic effect of several extracts of plant species used in traditional medicine such as *Selaginella nothohybrida, Selaginella lepidophylla* and *Tropaeolum majus* [31, 38].

The potassium-sparing effect of EFPA supposes also an action on the distal convoluted tubule and/or cortical collecting tube possibly by inhibiting the mineralocorticoid receptor, or with the exchanger Na^+^ / K^+^ by blocking sodium channels at the luminal membrane, respectively [25]. The present findings also indicate that *Phyllanthus amarus* could stimulate kidney function without causing an adverse effect on blood urea and creatinine [39].

## Conclusion

The present findings indicate diuretic properties of an ethanolic fraction of *Phyllanthus amarus* aqueous extract associated with significant urinary excretion of Na^+^ and water, which involves diuretic prostaglandins, supporting its use in traditional medicine as a potential remedy against hypertension. The diuretic activity of the *Phyllanthus amarus* extract might contribute to explain the hypotensive action shown previously in normal rabbits [14]. Further studies are needed to evaluate the effectiveness of EFPA in experimental models of hypertension, and to identify the bioactive compounds responsible for the diuretic properties.

## Additional files

Additional file 1: **Figure S1.** Urinary volumetric excretion induced by increasing doses of EFPA in rats. The urinary volumetric excretion values are expressed as a percentage of the initial hydric overload (50 mL/kg). Data are given as means ± SEM of 6 different experiments. Statistical analyses were assessed using two-way analysis of variance (ANOVA) followed by Bonferroni’s post- test. *p ˂ 0.001 versus control. (TIFF 216 kb)

Additional file 2: **Figure S2.** Inhibitory effect of indomethacin on urinary electrolyte excretion induced by EFPA in rats. Rats were treated over a 24 h period with a single dose of vehicle (control group), EFPA (10 mg/kg) or EFPA (10 mg/kg) + indomethacin (5 mg/kg, 1 h pretreatment) administered i.p. Data are given as means ± SEM of 6 different experiments. Statistical analyses were assessed using two-way analysis of variance (ANOVA) followed by Bonferroni’s post- test. *p < 0.05 versus control, and ^#^*p* < 0.05 versus EFPA. (TIFF 140 kb)

Additional file 3: **Figure S3.** Inhibitory effect of indomethacin on plasma electrolyte level induced by EFPA in rats. Rats were treated over a 24 h period with a single dose of either vehicle (control group), EFPA (10 mg/kg) or EFPA (10 mg/kg) + indomethacin (5 mg/kg, 1 h pretreatment) administered i.p. Data are given as means ± SEM of 6 different experiments. Statistical analyses were assessed using two-way analysis of variance (ANOVA) followed by Bonferroni’s post- test. *p < 0.05 versus control, and ^#^*p* < 0.05 versus EFPA. (TIFF 174 kb)

## Abbreviations

%: Percentage
Cl^-^: Chlorine
EFPA: Ethanolic fraction of *Phyllanthus amarus*
g: Gram
h: Hours
i.p: Intraperitoneal
K^+^: Potassium
L: Liter
mEq: Milliequivalents
Na^+^: Sodium
SEM: Standard error mean
UVE: Urinary volumetric excretion
