# Supplementary figures and images for: The acute diuretic effect of an ethanolic fraction of Phyllanthus amarus (Euphorbiaceae) in rats involves prostaglandins
Source: BMC Complement Altern Med. 2018 Mar 15;18:94. doi: 10.1186/s12906-018-2158-0 (PMC5856278; doi:10.1186/s12906-018-2158-0)

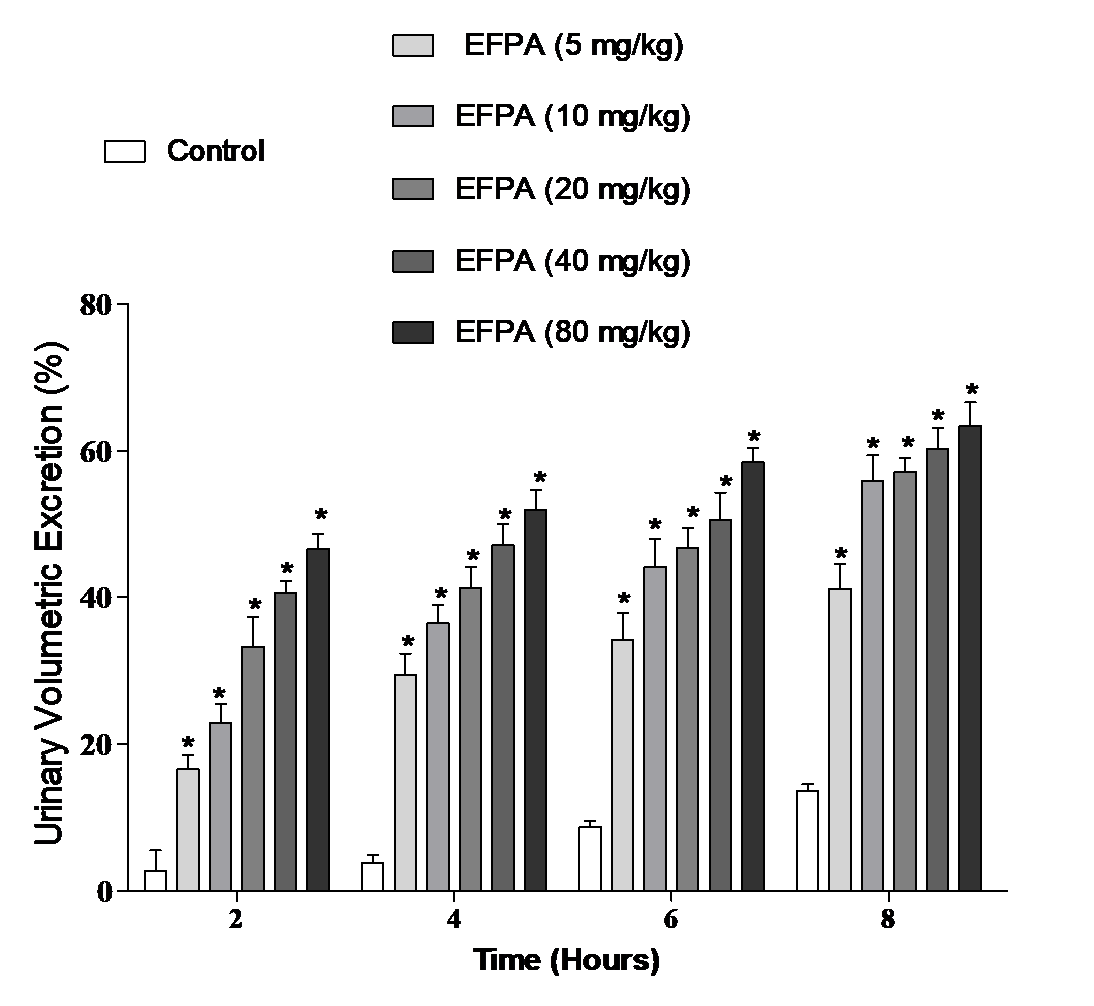

Supplement: Supplementary file 1 — Figure S1. Urinary volumetric excretion induced by increasing doses of EFPA in rats. The urinary volumetric excretion values are expressed as a percentage of the initial hydric overload (50 mL/kg). Data are given as means ± SEM of 6 different experiments. Statistical analyses were assessed using two-way analysis of variance (ANOVA) followed by Bonferroni’s post- test. *p ˂ 0.001 versus control. (TIFF 216 kb) [file 12906_2018_2158_MOESM1_ESM.tif]

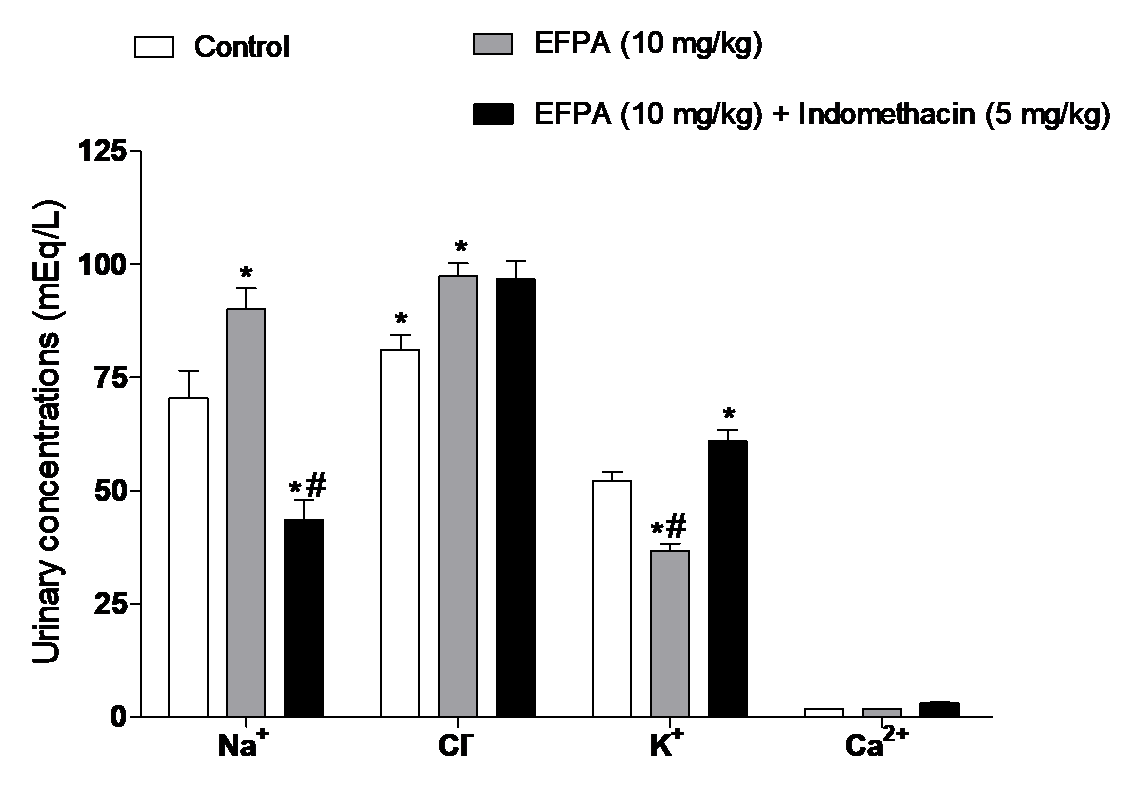

Supplement: Supplementary file 2 — Figure S2. Inhibitory effect of indomethacin on urinary electrolyte excretion induced by EFPA in rats. Rats were treated over a 24 h period with a single dose of vehicle (control group), EFPA (10 mg/kg) or EFPA (10 mg/kg) + indomethacin (5 mg/kg, 1 h pretreatment) administered i.p. Data are given as means ± SEM of 6 different experiments. Statistical analyses were assessed using two-way analysis of variance (ANOVA) followed by Bonferroni’s post- test. *p < 0.05 versus control, and #p < 0.05 versus EFPA. (TIFF 140 kb) [file 12906_2018_2158_MOESM2_ESM.tif]

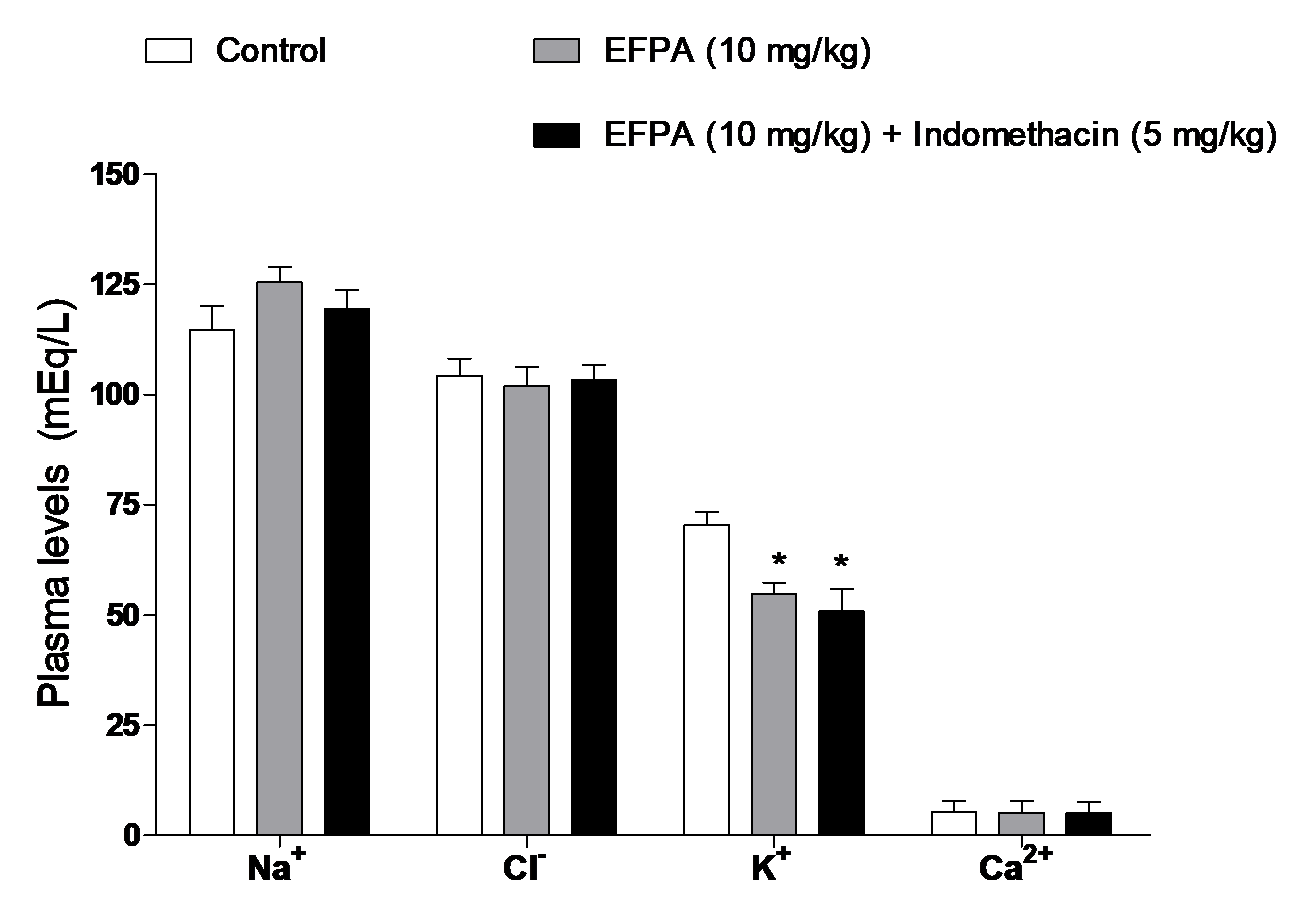

Supplement: Supplementary file 3 — Figure S3. Inhibitory effect of indomethacin on plasma electrolyte level induced by EFPA in rats. Rats were treated over a 24 h period with a single dose of either vehicle (control group), EFPA (10 mg/kg) or EFPA (10 mg/kg) + indomethacin (5 mg/kg, 1 h pretreatment) administered i.p. Data are given as means ± SEM of 6 different experiments. Statistical analyses were assessed using two-way analysis of variance (ANOVA) followed by Bonferroni’s post- test. *p < 0.05 versus control, and #p < 0.05 versus EFPA. (TIFF 174 kb) [file 12906_2018_2158_MOESM3_ESM.tif]
